# The Association of Maternal Exposure to Domestic Violence During Childhood With Prenatal Attachment, Maternal-Fetal Heart Rate, and Infant Behavioral Regulation

**DOI:** 10.3389/fpsyt.2018.00358

**Published:** 2018-08-07

**Authors:** Ana Sancho-Rossignol, Zoe Schilliger, María I. Cordero, Sandra Rusconi Serpa, Manuella Epiney, Petra Hüppi, François Ansermet, Daniel S. Schechter

**Affiliations:** ^1^Child & Adolescent Psychiatry Service, University of Geneva Hospitals, Geneva, Switzerland; ^2^Faculty of Health, Psychology and Social Care, Manchester Metropolitan University, Manchester, United Kingdom; ^3^Department of Obstetrics & Gynecology, University of Geneva Hospitals, Geneva, Switzerland; ^4^Developmental Pediatrics Service, University of Geneva Hospitals, Geneva, Switzerland; ^5^Department of Psychiatry, Faculty of Medicine, University of Geneva, Geneva, Switzerland; ^6^Department of Child & Adolescent Psychiatry, New York University Langone Medical Center and School of Medicine, New York, NY, United States

**Keywords:** childhood trauma, domestic violence, emotion regulation, prenatal attachment, infant behavior, parenting, perinatal mental health

## Abstract

Human and animal models suggest that maternal hormonal and physiological adaptations during pregnancy shape maternal brain functioning and behavior crucial for offspring care and survival. Less sensitive maternal behavior, often associated with psychobiological dysregulation and the offspring's behavioral and emotional disorders, has been observed in mothers who have experienced adverse childhood experiences. Strong evidence shows that children who are exposed to domestic violence (DV) are at risk of being abused or becoming abusive in adulthood. Yet little is known about the effect of childhood exposure to DV on the expecting mother, her subsequent caregiving behavior and related effects on her infant. Thus, the present study examined the association of maternal exposure to DV during childhood on prenatal maternal attachment, maternal heart rate reactivity to an infant-crying stimulus and post-natal infant emotional regulation. Thirty-three women with and without exposure to DV during childhood were recruited during the first trimester of pregnancy and followed until 6-month after birth. The Maternal Antenatal Attachment Scale (MAAS) was used to measure prenatal attachment of the mother to her fetus during the second trimester of pregnancy, maternal and fetal heart rate reactivity to an infant-crying stimulus was assessed at the third trimester of pregnancy, and the Infant Behavior Questionnaire-Revised (IBQ-R) was used to assess infant emotional regulation at 6-months. Results showed that pregnant women that were exposed to DV during childhood had a poorer quality of prenatal attachment of mother to fetus, regardless of whether they also experienced DV during adulthood. In addition, maternal exposure to DV during childhood was associated with increased maternal heart rate to infant-crying stimulus and worse infant emotional regulation. These findings highlight the importance of prenatal screening for maternal exposure to DV during childhood as a risk factor for disturbances in the development of maternal attachment, dysfunctional maternal behavior and emotion dysregulation.

## Introduction

The experience of domestic violence (DV) has been found to have a likely profound negative impact both on maternal psychological functioning and on the mother-infant relationship (1–3). One study to date has shown that even prior to birth, expectant mothers with a history of interpersonal trauma exposure (i.e., childhood physical and/or sexual abuse and/or adult sexual and/or physical assault including DV) had a poorer quality of prenatal attachment of mother to her fetus on the Maternal Antenatal Attachment Scale (4) than those mothers who reported having no interpersonal trauma exposure (5).

Growing evidence supports that the experience of adverse childhood experiences or “ACES,” such as maltreatment, without specific study of childhood exposure to DV known to date, can adversely influence maternal behavior during adulthood (6). In spite of over a decade of mounting evidence of the long-lasting physical and mental health effects of ACES (7, 8), and possible transgenerational transmission (9, 10), there is still a dearth of published research investigating the effects of women's past history of these ACES on pregnancy and fetal development. As maternal and fetal physiology are closely related, maternal physiological responses to external stimuli may affect the fetus' physiological state and reactivity such as through fetal programming (11). Emerging evidence suggests that maternal ACES may also influence fetal neurobehavioral development. For example, Cordero et al. (12) found that during the third trimester of pregnancy, the fetuses of pregnant adolescents with a history of emotional abuse during childhood had reduced resting heart-rate variability (i.e., decreased parasympathetic activity). And yet another study demonstrated an association between maternal exposure to childhood maltreatment and lower cortical gray matter in their newborns (13).

The mother-infant relationship is an asymmetric, bidirectional system. While maternal behavior through the lens of a more maturely developed complex neurobiological system influences infant physiology and behavior, infant behavior (e.g., crying) also induces a maternal physiological response that has been suggested to function as a “preparation for action” in terms of maternal behavior (14). Of note, children of mothers who experienced intimate partner violence during pregnancy and/or postnatally have altered hypothalamus-pituitary-adrenal axis and autonomic stress responses to relational stressors such as mother-child separation (15–17) and are at risk of developing behavioral and emotional disorders (16).

Even though exposure to DV during childhood has been shown in and of itself to be a potent risk factor for future involvement in DV relationships (18), no studies to our knowledge have specifically examined maternal exposure to DV during childhood related to maternal-fetal autonomic nervous system functioning and infant behavioral development. Furthermore, although the quality of maternal prenatal attachment of mother to fetus is a robust predictor of the quality of postnatal mother-infant attachment and mother-child interaction (19, 20), little is known about the specific effects of maternal exposure to DV during childhood on prenatal attachment of mother to fetus.

We thus conducted a study to test the following hypotheses: mothers who report having been exposed to DV during childhood would show: (1) Reduced maternal prenatal attachment to the fetus, as well as (2) Altered maternal and fetal heart rate as a marker of the sympathetic nervous system activity in response to a crying infant stimulus. Additionally, (3) 6 months after birth, infants of mothers with histories of childhood DV exposure would show greater behavioral dysregulation.

## Methods

### Participants

**Figure 1:**
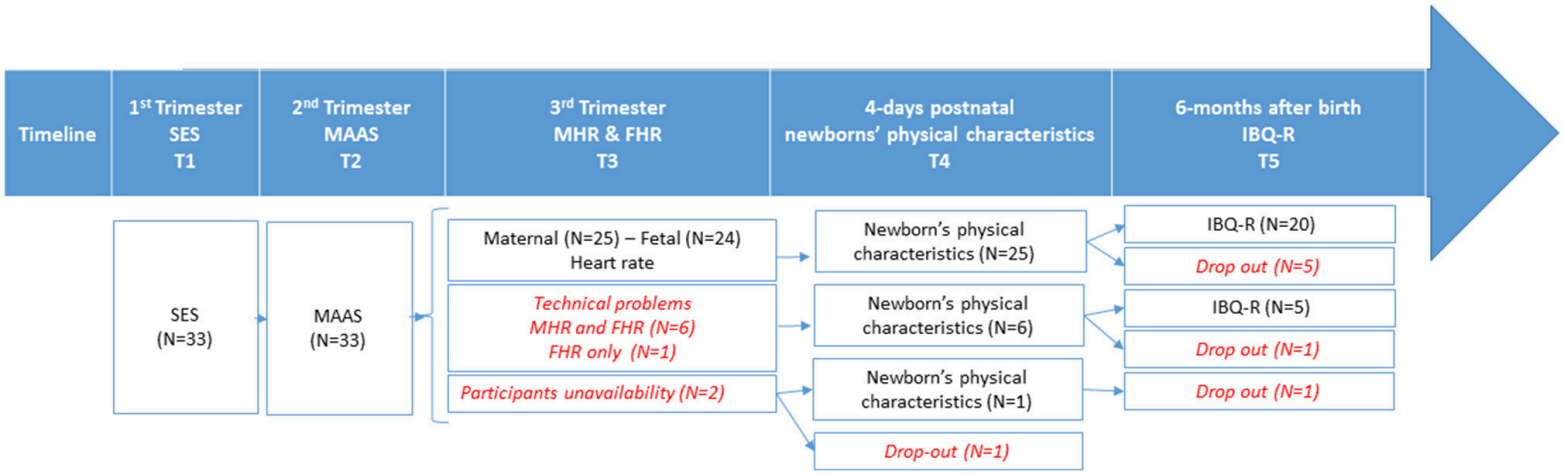
Flow chart depicting the number of participants at each study collection point including drop-outs and excluded participants. Additionally, the numbers of excluded participants and drop-outs are depicted. IBQ-R, Infant regulatory behavior; MAAS, Maternal Antenatal Attachment Scale; MHR, Maternal heart rate; FHR, Fetal heart rate; SES, Largo socio-economic status Index.

This study is part of a longitudinal project that investigates the influence of prenatal stress on infant psychosocial development. Here we presented data of 33 pregnant women recruited at their first visit (January 2012-January 2015) to the Geneva Obstetric Clinic of the University Hospital (10–12 weeks of gestation) and followed until 6 month after birth. Inclusion criteria for the data collected to be included in the analyses required that mothers to have had a full-term pregnancy without complications requiring medical intervention. Before delivery, one of the women moved to another country and abandoned the study. Thirty-two infants (41% male) were all born at term (M = 38.84 weeks; SD = 1.44) and had a normal birth weight (M = 3.23 kg; SD = 0.51) and 5-min Apgar scores (M = 9.84; SD = 0.45). At the 6-month visit, six participants dropped out reducing the postnatal sample to 25 subjects. A flow chart with information of participants' sample at each period of data collection is illustrated in Figure 1. Participant demographics are listed in Table 1. For their participation in the study, the participants were compensated with 50 Swiss Francs per session. The study was conducted in accordance with the 1964 Helsinki declaration and its later amendments, and was approved by the ethics committee of the University of Geneva Hospitals and Faculty of Medicine.

**Table 1:** Socio-economic characteristics of study participants at multiple data collection time points (before giving birth: T1 = 12–14, T2 = 20–24, and T3 = 30–34 weeks of gestational age; after giving birth: T4 = day 4th and T5 = 6 months).

	**All cohort (T1/T2)**	**Prenatal cohort with MAAS and HR (T3/T4)**	**Postnatal cohort with MAAS and IBQ-R (T5)**
	**(*N* = 33)**	**(*N* = 25)**	**(*N* = 25)**
	***M (SD)***	***M (SD)***	**M (SD)**
Maternal Age (years)	29.6 (7.0)	30.3 (4.8)	31.1 (7.1)
Largo SES Index (score)	7.4 (2.5)	7.3 (2.4)	7.2 (2.5)
Gestational age at term (weeks)	38.84 (1.4)	39.16 (.9)	38.76 (1.59)
**Ethnic Group**:	***N*** **(%)**	***N*** **(%)**	***N*** **(%)**
European	22 (63)	14 (56)	15 (60)
African	5 (14)	4 (16)	4 (16)
Latin American	8 (23)	7 (28)	6 (24)
**Marital Status:**			
Single	11 (33)	8 (32)	6 (24)
Married	21 (64)	16 (64)	18 (72)
Divorced	1 (3)	1 (4)	1 (4)
**M-DV-Ch**	11 (31%)	9 (36%)	8 (32%)
**Newborns:**	***N*** = **32** **N (boys %)**	***N*** = **24** **N (boys %)**	***N*** = **24** **N (boys %)**
Sex (boys+girls)	13 + 19 (41)	11 + 14 (44)	11 + 14 (44)
	***M (SD)***	***M (SD)***	**M (SD)**
Weight at birth (g)	3235 (508)	3304 (466)	3280 (524)
Apgar 5-min (score)	9.84 (.45)	9.88 (.33)	9.84 (.47)

*IBQ-R, Infant regulatory behavior; HR, heart rate, M-DV-Ch, maternal exposure to domestic violence during childhood; MAAS, Maternal Antenatal Attachment Scale; SES, Largo socio-economic status Index*.

### Measures

#### Maternal exposure to domestic violence during childhood

Maternal exposure to domestic violence during childhood (M-DV-Ch) was assessed during the first visit via the Geneva Prenatal Stress Questionnaire (21), a screening measure that probes for a history of maternal stressful life-events before and during pregnancy. In order to test our *a-priori* hypothesis concerning the association of M-DV-Ch with target variables, mothers were asked the following question: “*Did you experience domestic violence at home in your childhood (up to age 16)?”* and were then invited to describe the events if the answer was affirmative. However, no further elaboration or other specific questions about the events was requested so as to avoid upsetting the participants with memories of traumatic events. Eleven participants declared to have been exposed to domestic violence during childhood. The extent of detail regarding the experience of domestic violence differed greatly between participants, though in most the information provided was very scarce, with no specific information regarding period of exposure or DV severity.

#### Maternal prenatal attachment to the fetus

The quality of prenatal attachment of mother to her fetus was measured using the Maternal Antenatal Attachment Scale (MAAS) during the second trimester follow-up session. The MAAS (4) is a 19-item self-report questionnaire reflecting the mother's emotional connection to the fetus (22), with two subscales: (1) quality of attachment, and (2) time spent in “attachment mode,” which represents the intensity of “primary maternal preoccupation” (23, 24) or psychological investment that the pregnant woman experiences based on her feelings, behaviors and attitudes toward the fetus (25, 26). The addition of both subscales provides the global score. The MAAS is a validated measure that has shown high levels of internal consistency (4). Internal consistency in our study was adequate (Cronbach alpha = 0.71).

#### Maternal and fetal cardiac activity

Maternal and fetal electrocardiograms were recorded simultaneously using the MONICA AN24 wireless fetal monitor (Monica Healthcare, Nottingham, UK) during the third trimester of pregnancy. This portable device permits a non-invasive recording of maternal and fetal cardiac electrical signals and uterine activity using five abdominally-placed electrodes. Data collected were transferred to a laptop and analyzed using the manufacturer's software that allows manual editing for artifacts and to extract maternal and fetal beat-to-beat heart rate (HR). The latter provided HR in bpm in 0.25 s epochs as well as beat-to-beat R–R intervals in 0.25 s epochs. The average maternal and fetal HR measured during relaxation (3 min) and exposure to baby crying auditory stimulus (2 min) was calculated. Because of technical problems during data acquisition (*N* = 6) or participants' unavailability (*N* = 2), only 25 women underwent cardiac monitoring. Results for fetal heart rate (FHR) are reported for only 24 fetuses, as the FHR data of one participant was excluded when no fetal HR signal was obtained due to an artifact.

#### Infant regulatory behavior

Infant regulatory behavior was measured at 6-months of age with the French translation of the Infant Behavior Questionnaire-Revised Short Form version (IBQ-R-SF) (Cascales, unpublished). IBQ-R-SF contains 91-items grouped in 14-scales (27). Parents are asked to rate the frequency that their infant engaged in specific day-to-day behaviors in the prior week (1 = never to 7 = always). The IBQ-R-SF is a validated measure that has good psychometric properties for the assessment of infant regulatory behavior: internal consistency (Cronbach's alphas typically range from 0.70 to 0.90) and good inter-rater reliability (28, 29). Internal consistency in our study was adequate (Cronbach alpha = 0.71).

In order to test our hypothesis that infants of mothers with histories of childhood exposure to DV would have poor emotional and behavioral regulatory capacities, we decided *a-priori* to exclusively use the Orienting/Regulation Scale to limit comparisons given our small sample size. According to IBQ-R SF scoring, this scale is the overall score of three subscales: (1) Duration of orientation (i.e., infant's attention to and/or interaction with objects), (2) High Intensity Pleasure (i.e., amount of pleasure or enjoyment related to high stimulus intensity and complexity), and (3) Soothability (i.e., infant's reduction of fussing, crying or distress when soothing techniques are used by the caregiver).

#### Socio-demographic variables

Familial socio-economic status (SES) was calculated using the Largo SES Index (30), a well-validated measure that adds maternal educational level and paternal profession and provides a global score ranging from 2 (the highest score) to 12 (the lowest score).

#### Maternal psychological assessment

In order to gather general information regarding their psychological mental state, participants were asked to complete three questionnaires to assess state anxiety, depression, and psychopathological symptoms.

State anxiety was measured with the French adaptation of the State-Trait Anxiety Inventory [STAI; (31, 32)], a reliable and valid instrument to assess anxiety in general population and in pregnancy (33). Participants were asked to complete the State scale during their visits at the first, second and third trimester. State anxiety scale contains 20 items asking the participants to rate on a Likert scale (1, not at all to 4, very much) how they felt at that particular time. Scores were averaged for the three trimesters and compared between groups (pregnant women exposed to DV during childhood vs. non-exposed pregnant women) across the three subsamples (T1/T2, T3/T4, T5).

Depressive symptoms were evaluated at each trimester during pregnancy with the French version of the Edinburgh Postnatal Depression Scale [EPDS; (34)]. The EPDS has a high reliability to assess depressive symptoms during pregnancy. The EPDS is a short scale of 10 items assessing mood during the last 7 days. Participants rate the items on a 4-point Likert scale (0–3), with higher scores indicating higher depressive symptoms. Scores were averaged for the three trimesters and compared between groups (pregnant women exposed to DV during childhood vs. non-exposed pregnant women) across the three subsamples (T1/T2, T3/T4, T5).

The Symptom Checklist-90-Revised [SCL-90-R; (35, 36)] is a widely-used 90-item self-reported inventory designed to evaluate a broad range of psychological problems and symptoms of psychopathology using nine psychiatric symptoms, including somatization, obsessive-compulsive disorder, interpersonal sensitivity, depression, anxiety, hostility, phobic anxiety, paranoid ideation, and psychoticism. Given the inclusion of items regarding somatic symptoms that can overlap with pregnancy-related somatic changes, participants were asked to complete the questionnaire only at the first visit (T1). Each item is graded on a 5-point scale of distress (0, never to 4, extremely). The SCL-90-R has demonstrated good reliability and validity (37). Cronbach's alpha reliability coefficient was 0.944.

### Procedure

This study is part of a longitudinal project that investigates the influence of prenatal stress on infant psychosocial development. Here we presented data of 33 pregnant women recruited at their first visit to the Geneva Obstetric Clinic of the University Hospital (10–12 weeks of gestation) and followed until 6 month after birth. Follow-up sessions were performed once at each trimester of pregnancy (respectively at T1 = 12–14, T2 = 20–24, and T3 = 30–34 weeks of gestational age); and two postnatal visits, the fourth day after birth (T4) and 6-months afterwards (T5). Oral and written informed consent was obtained from all participants.

The data collected at the hospital was as follows (Figure 1): M-DV-Ch exposure, STAI, EPDS, SCL-90, and SES during the first session (T1), MAAS, STAI and EPDS at the second session (T2), and maternal and fetal HR, STAI and EPDS during the third session (T3). In order to perform the electrocardiogram (ECG) and measure maternal and fetal HR, participants were asked not to exercise or drink for 30-min prior the session, and to avoid speaking or making any unnecessary movement during the ECG. The session was scheduled after a routine ultrasound; and the information about the position of the fetus was used to place the electrodes. Once the participant had reclined comfortably on the bed, the clinician explained the procedure briefly and placed the ECG electrodes and the earphones used to deliver the auditory stimulus. Participants were asked to relax for 3-min before the baby crying stimulus (2-min duration) was played through the earphones. Stimulus onset and offset was signaled using an event marker.

The first postnatal session took place at the hospital 4 days after delivery (T4) and information related to delivery and wellbeing of the mother and newborn and physical characteristics of the newborn were gathered. The last postnatal session took place 6-months after delivery (T5), and mothers were asked to complete the IBQ-R-SF.

### Statistical analysis

Data were analyzed using the Statistical Package for Social Sciences Software (SPSS, version 24.0) for Windows. Normal distribution of continuous variables was assessed by Shapiro-Wilk tests. Descriptive statistics for all socio-demographic and other variables were calculated and expressed appropriately in percentages, mean values (M) and standard deviation (SD), or median (Mdn) and mean rank. Non-normally distributed data were analyzed using Mann–Whitney-*U* tests for between-subject comparisons (non-exposed vs. exposed to domestic violence during childhood), and Spearman's non-parametric correlations (r_s_) was used to test associations between maternal and fetal HR. Effect size, *r*, was calculated by dividing Z by the square root of N. We used Pearson's X^2^ to compare nominal variables (marital status, ethnic group, parity and infants' gender) between the groups, and φ for effect size. For all analyses, *p* < 0.05 was considered statistically significant. *Post-hoc* power analysis for MAAS, maternal and fetal HR and IBQ-R were performed using G^*^Power 3.1.9.2 (Dusseldorf, Germany).

## Results

### Descriptive data

Data collected at the different time points differed in the constitution of the samples (i.e., not all the participants were able to attend all the sessions). Table 1 details sample characteristics at each of the time points (T1/T2, N = 33; T3/T4, N = 25/24; T5, N = 25), including maternal age, SES, gestational age at term, ethnic group, marital status, exposure to domestic violence during childhood, and newborns' sex, weight and Apgar 5-min scores.

The group of women who were exposed to DV in childhood did not differ in a statistically significant way from the group of women without such exposure in terms of the following measures: maternal age, SES, gestational age at term, weight at birth, Apgar 5-min scores, state anxiety (STAI), depression symptoms (EPDS) or psychopathology (SCL-90-R (Table 2); nor on any of the socio-demographic variables, parity status or newborns' sex (Table 3; distribution of cases per variable is presented in Table 4).

**Table 2:** Mann-Whitney analyses of the sociodemographic and newborns data comparing the group of pregnant women exposed to domestic violence during childhood with the non-exposed group.

**Sample**	**Group**	**Statistic**	**Maternal age (years)**	**Largo SES Index**	**Gestational age at term (weeks)**	**Weight at birth (g)**	**Apgar 5-min (score)**	**STAI $\left(\bar{X}\right)$**	**EPDS $\left(\bar{X}\right)$**	**SCL-90- R (score)**
T1/T2	Non-exposed	*Mdn*	30	8.00	39	3430	10	31	7.33	48.5
		Mean Rank	18.05	17.41	16.50	17.5	16.19	16.64	16.62	17.36
	M-DV-Ch	*Mdn*	24	8.00	39	3380	10	35.7	7.5	50.0
		Mean Rank	14.91	16.18	16.50	14.59	17.09	17.73	16.27	16.27
		N	33	33	32	32	32	33	32	33
		U	98	112	115.5	94.5	122	129	113	113
		Z	−0.88	−0.36	0	−0.83	0.45	0.31	−0.1	−0.31
		*p*	0.40	0.75	1	0.41	0.82	0.78	0.94	0.78
		*r*	−0.15	−0.06	0.00	−0.15	0.08	0.05	−0.02	−0.05
T3/T4	Non-exposed	*Mdn*	30.5	8.00	39	3425	10	31	5.67	48.5
		Mean Rank	14.03	11.91	12.69	13.62	12.94	12.47	12.17	13.25
	M-DV-Ch	*Mdn*	24	8.00	39	3380	10	31.67	6.0	50
		Mean Rank	11.17	14.94	13.56	11.89	13.11	13.94	13.06	12.56
		N	25	25	25	25	25	25	24	25
		U	55.5	89.5	77	62	73	80.5	72.5	68
		Z	−0.94	1.04	0.30	−0.57	0.1	0.48	0.3	−0.23
		*p*	0.36	0.33	0.8	0.6	1	0.64	0.77	0.85
		*r*	−0.19	0.21	0.06	−0.11	0.02	0.1	0.06	−0.05
T5	Non-exposed	*Mdn*	30	8.00	39	3430	10	31	6.67	55
		Mean Rank	13	12.65	12.88	13.74	12.29	12.50	12.53	13.47
	M-DV-Ch	*Mdn*	34.5	8.00	39	3380	10	36.33	6.85	50.50
		Mean Rank	13	13.75	13.25	11.44	14.50	14.06	12.44	12.00
		N	25	25	25	25	25	25	24	25
		U	68	74	70	55.5	80	76.5	63.5	60
		Z	0	0.36	0.12	−0.73	1.24	0.49	−0.03	−0.47
		*p*	1	0.75	0.93	0.47	0.51	0.63	0.98	0.67
		*r*	0.00	0.07	0.02	−0.15	0.25	0.1	−0.01	−0.09

*The analyses are performed for each reference sample (before giving birth: T1, 12–14; T2, 20–24; T3, 30–34 weeks of gestational age; after giving birth: T4, day 4th; T5, 6 months)*.

**Table 3:** Pearson's X ^2^ analyses of the marital status, ethnic group and infants' gender performed for each reference sample (before giving birth: T1 = 12–14, T2 = 20–24, and T3 = 30–34 weeks of gestational age; after giving birth: T4 = day 4th and T5 = 6 months).

**Sample**	**Statistic**	**Ethnic group**	**Marital status**	**Parity**	**Sex**
T1/T2	N	33	33	33	32
	X	0.69	0.896	1.517	1.26
	df	2	2	1	1
	*p*	0.71	0.64	0.22	0.72
	Φ	0.14	0.164	0.21	0.198
T3/T4	N	25	25	25	25
	X	0.663	0.59	2.71	0.65
	df	2	2	1	1
	*p*	0.72	0.75	0.10	0.42
	Φ	0.16	0.15	0.33	0.161
T5	N	25	25	25	25
	X	0.11	1.5	1.72	0.2
	df	2	2	1	1
	*p*	0.95	0.47	0.19	0.65
	Φ	0.07	0.25	0.26	0.09

**Table 4:** Distribution of number of cases in each group for each category: ethnic group, marital status and newborns' sex.

**Variable**	**Categories**	**T1/T2**		**T3/T4**		**T5**	
		**Non-exposed (*N* = 22/21)**	**M-DV-Ch (*N* = 11)**	**Non-exposed (*N* = 16)**	**M-DV-Ch (*N* = 9)**	**Non-exposed (*N* = 17)**	**M-DV-Ch (*N* = 8)**
Ethnic Group	European	13	8	8	6	10	5
	African	4	1	3	1	3	1
	Latin American	5	2	5	2	4	2
Marital Status	Single	8	3	5	3	5	1
	Married	13	8	10	6	11	7
	Divorced	1	0	1	0	1	0
Parity	Primiparity	13	4	9	2	9	2
	Multiparity	9	7	7	7	8	6
Newborns' Sex	Boys	9	4	8	3	8	3
	Girls	12	7	8	6	9	5

### Prenatal attachment

**Figure 2:**
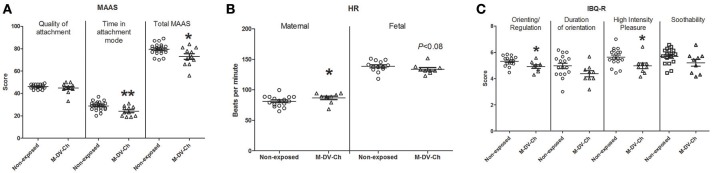
Scatter plot showing **(A)** scores in the Maternal Antenatal Attachment Scale (MAAS) administered during the 2nd trimester session; **(B)** Maternal and fetal heart rate (HR) during infant crying stimuli recorded during the 3rd trimester; and **(C)** scores in the Infant Behavior Questionnaire-Revised (IBQ-R) at 6 months after birth. M-DV-Ch, maternal exposure to domestic violence during childhood. Mann-Whitney *U* test was used for testing level of significance (^*^p < 0.05; ^**^p < 0.01).

Prenatal attachment was assessed at the second trimester follow-up session using the MAAS. Significant differences between groups were found on attachment mode subscale and total MAAS (Table 5 and Figure 2A), pregnant women exposed to domestic violence during childhood (*N* = 11) spent less time in attachment mode (*p* < 0.01) and had lower total MAAS scores compared to the non-exposed group (*N* = 22; *p* < 0.05). No differences were found between groups on quality of attachment of mother to fetus.

**Table 5:** Mann-Whitney analyses of MAAS, maternal and fetal HR and IBQ-R-SF comparing the group of pregnant women exposed to domestic violence during childhood with the non-exposed group (^*^p < 0.05; ^**^p < 0.01).

**Measure**	**Subscales and Total**	**Non-exposed *Mdn*** ***Mean Rank***	**M-DV-Ch *Mdn*** ***Mean Rank***	**N**	***U***	***Z***	***p***	***r***	***power***
MAAS	Attachment mode^**^	30 20.05	23 10.91	33	54	−2.57	0.01	0.45	0.13
	Quality of attachment	46 17.77	45 15.45	33	104	−0.66	0.51	0.11	0.53
	Total MAAS scores^*^	80 19.52	73 11.95	33	65.5	−2.12	0.04	0.37	0.20
**Measure**	**Event**								
MHR	Infant crying stimulus^*^	81.71 10.69	88.90 17.11	25	109	2.09	0.04	0.42	0.20
	Relaxation	83.95 11.56	89.5 15.56	25	95	1.3	0.19	0.26	0.31
FHR	Infant crying stimulus	137.75 14.53	131.58 9.11	24	37	−1.82	0.08	0.37	0.24
	Relaxation	137.25 13.40	135.90 11.00	24	54	−0.8	0.42	0.16	0.47
**Measure**	**Subscales and Total**								
IBQ-R-SF	High intensity pleasure^*^	5.57 15.15	5.0 8.44	25	31.5	−2.13	0.04	0.43	0.20
	Duration of orientation	4.83 14.76	4.25 9.25	25	38	−1.76	0.09	0.35	0.24
	Soothability	5.86 14.76	5.21 9.25	25	38	−1.75	0.09	0.35	0.24
	Infant's Orienting/ Regulation IBQ-R scale^*^	5.5 15.35	4.97 8.00	25	28	−2.33	0.02	0.47	0.15

### Cardiac reactivity

During the third trimester of pregnancy, maternal and fetal HR was recorded during a short period of relaxation and exposure to an infant crying stimulus. Compared to the non-exposed group (*N* = 16), pregnant women that were exposed to domestic violence during childhood (*N* = 9) showed increased HR during the infant stimulus (Table 5 and Figure 2B; *p* < 0.05). Fetal HR during the infant crying stimulus tended to be lower in M-DV-Ch (*N* = 9) than in the non-exposed group (*N* = 15) (Table 5 and Figure 2B; *p* = 0.08). Maternal and fetal HR did not differ significantly between groups during relaxation.

Maternal and fetal HR showed a negative significant correlation during the infant crying stimulus (r_s_ = −0.54, *p* < 0.01, *N* = 24) and a tendency to significance during the relaxation/resting time (r_s_- = −0.38, *p* < 0.07, *N* = 24).

### Maternal report of infant behavioral regulation

Compared to the non-exposed group, M-DV-Ch infants received significantly lower scores in the infant's Orienting/Regulation IBQ-R scale (Table 5 and Figure 2C; *p* < 0.05) and on the Orienting/Regulation's subscales though the scores were statistically significant only for the “high intensity pleasure” subscale (*p* < 0.05); “duration of orientation” subscale and “soothability” subscale were not statistically significant.

## Discussion

The present study addressed the associations among maternal exposure to DV during childhood and prenatal attachment of mother to fetus, maternal and fetal HR reactivity during pregnancy, and infant behavioral regulation at 6-months of age. Results showed that maternal exposure to DV during childhood was associated with reduced prenatal attachment of mother to fetus, increased prenatal maternal HR reactivity to crying-infant stimulus (and inversely, reduced fetal HR reactivity—albeit at a trend-level of significance), and poorer infant behavioral regulation.

In the present study, maternal exposure to DV during childhood (i.e., exposure to negative early relational experiences) was associated with a poorer maternal quality of attachment to the fetus and with less time spent in attachment mode and less maternal preoccupation with the fetus. The present study's findings together with those of Schwerdtfeger and Goff (5), indicate that maternal exposure to DV during childhood is significantly associated with the quality of prenatal attachment of mother to fetus. This in and of itself is important since the quality of prenatal attachment has been shown to be a predictor of the quality of postnatal mother-infant attachment and interactive behavior, including for caregivers, the degree of maternal sensitivity (38).

Furthermore, the present study found that pregnant women who experienced exposure to DV during childhood displayed increased HR in reaction to a crying infant stimulus. Interestingly, it has been reported that mothers with a strong physiological reaction to infant crying are more likely to present greater irritation and aversive or harsh parenting behavior to infant crying stimuli (39). Mothers with this vulnerability have also been reported to have greater difficulty in discerning between different types of cries, i.e., hunger, pain, fatigue, or fear (40). Maternal autonomic hyperactivity in response to crying infant has been negatively correlated with the degree of maternal sensitivity and has been associated with a potential risk for intergenerational transmission of maltreatment and interpersonal violence exposure (41, 42).

The present study additionally found a significant negative correlation between maternal and fetal HR in response to crying infant stimulus, and a trend toward low FHR during exposure to the crying infant stimulus among mothers exposed to DV during childhood. Though the relationship between maternal and fetal HR involves several factors including fetal behavioral state and level of maturity, maternal stress responses and anxiety during pregnancy have been related to reduced FHR (43) and reduced feto-placental volume blood flow in third trimester of pregnancy (44).

The present study also found that infants of mothers exposed to DV during childhood appear to show greater difficulty with emotion regulation and arousal by the age of 6 months. Consistent with this finding is that of Enlow et al. (45) who found maternal lifetime trauma exposure to be associated with infant negative affectivity at 6-months. These results are consistent with other studies in which mothers with a history of early adverse experiences such as lifetime history of DV exposure and childhood maltreatment present difficulties in discerning the nature of infant distress (46). The latter has been associated with insensitive responsiveness and increased risk for subsequent maltreatment and exposure to violence regarding their own children (47). Similar findings have been reported for mothers with DV-related post-traumatic stress disorder (PTSD) who have been shown to have increased difficulty identifying emotions and present impaired sensitive maternal behavior compared to mothers without PTSD (48). Of interest for future research, this study did not measure maternal trauma-related psychopathology before or after birth, which may be a mediating and/or moderating factor for possible effects on mother-fetal/infant attachment and physiologic response. There are a number of other possible mediating and/or moderating factors for such effects, which also would be important to study and for which one would in future research want to control. These include, among others, factors such as the following: general prenatal stress (49), social support (50), poverty (51), and postnatal infant reactivity (52), the latter which has been shown to moderate the association between maternal prenatal depression and infant sleep postnatally.

Future research might also consider potential associations among the quality of maternal mental representations of primary attachment figure(s), the quality of the mother-fetus/infant attachment relationship, and mother-infant interactive behavior (53–55).

Limitations of this study, beyond its small sample size, also include the use of a close-ended “yes/no” screening question concerning DV exposure during childhood and no pressure to elaborate and provide specific information such as age of exposure, frequency, and intensity, important factors in terms of effects on the developing brain (56). And yet for clinical purposes, we have been able to show that even such a simple yes/no question in a rapid primary-care clinical screening encounter, for example, it can yield important risk indicators requiring follow-up. Furthermore, this closed-ended trauma screener is retrospective and involves all limitations pertinent to retrospective life-events measures. Another important limitation of this study is its reliance on maternal report measures of child behavior. Since maternal report of child behavior may be biased by maternal perception that is influenced by maternal psychopathology and/or a history of a mother's own attachment disturbance, the use of a clinician-rated observational measure of behavior would be an important component to include in future research. An additional limitation of the study was the lack of a measure of maternal posttraumatic stress disorder, which is frequently associated with childhood DV exposure (48). Another important limitation of the study related to the small sample mentioned above is the need of awareness of the actual significance of the findings when performing multiple comparisons. Given the lack of consensus of how to estimate the number of comparisons (57), multiple comparison corrections were included, though we provided statistical values (effect size and power) for all the analyses in order to provide information regarding the magnitude of the findings.

Clinical implications of the findings of this study highlight the importance of assessing pregnant women's history of early and subsequent adverse life-events and the quality of attachment to their fetus as possible risk factors for postnatal disturbances. This risk can be reduced by a preventive intervention during pregnancy with focus on (i) reducing physiological arousal to a crying infant stimulus, and (ii) improving the quality of early attachment of mother to fetus. In the early postnatal period, interventions like mentalization-based treatments (58) or clinician-assisted video feedback exposure (48) could be implemented to support these mothers at risk, and prevent mother-infant attachment disturbances and intergenerational transmission of violence and trauma (59).

## Author contributions

AS-R, MC, SR, ME, PH, FA, and DS conceived and designed the study. AS-R, MC, ZS, and DS together wrote the first drafts of the manuscript. AS-R and MC collected data and prepared all the data for analyses. AS-R, MC, and ZS performed the statistical analysis. All authors critically revised several versions of the manuscript and approved the final version.

### Conflict of interest statement

The authors declare that the research was conducted in the absence of any commercial or financial relationships that could be construed as a potential conflict of interest.

## Abbreviations

ACE: adverse childhood experience
DV: domestic violence
ECG: electrocardiogram
FHR: fetal heart rate
HR: heart rate
IBQ-R-SF: Infant Behavior Questionnaire-Revised Short Form version
MAAS: Maternal Antenatal Attachment Scale
M-DV-Ch: Maternal exposure to domestic violence during childhood
SES: Socio-economic status.
